# Warm and Cold Bathing

**Published:** 1858-02

**Authors:** 


### WARM AND COLD BATHING.

THE safest plan is the best. This is as true in medicine as in any other department of human life. We had far rather allow a patient to die without medicine, than kill him by an inopportune dose.

Another sentiment of ours is, to try the more agreeable remedy first. A dose of medicine which is palatable to a patient will do him more good, other things being equal, than one which is repulsive. That a warm bath is safer than a cold one, no intelligent person can call in question; that it is more soothing, even in a burning fever, is without doubt; while its agency in "cooling" any diseased part of the body, will be admired by every one who chooses to make the experiment: it does all the good, without the shock of cold water, which is even painful to some, and insupportable to not a few. A forcible writer has observed, with a great deal of truth

"The warm bath is a grand remedy, and will cure the most virulent of diseases. A person who may be in fear of having received infection of any kind, as, for instance, having visited a fever patient, should speedily plunge into a warm bath, suffer perspiration to ensue, and then rub dry, dress securely to guard against taking cold, and finish off with a cup of strong tea, by the fire. If the system has imbibed any infectious matter, it will certainly be removed by this process, if it be resorted to before the infection has time to spread over the system. And even if some time has elapsed, the drenching perspiration that may be induced in a hot bath will be pretty sure to remove it."

*Life Illustrated*, the staunch advocate of cold water, says, with great truth, " whatever may be the merits of the judicious application of water, cold or hot, its improper use will certainly do mischief, and may destroy life."

Our advice to all then is, that the application of even water, as a remedial means, should be under the supervision of a sound judgment, or an experienced physician.

